# Success Factors in the FIFA 2018 World Cup in Russia and FIFA 2014 World Cup in Brazil

**DOI:** 10.3389/fpsyg.2021.638690

**Published:** 2021-03-09

**Authors:** Hannes Lepschy, Alexander Woll, Hagen Wäsche

**Affiliations:** Institute of Sports and Sports Science, Karlsruhe Institute of Technology (KIT), Karlsruhe, Germany

**Keywords:** match analysis, performance analysis, performance indicators, soccer, sport analytics

## Abstract

Research on success factors in football focusing on national teams is sparse. The current study examines the success factors during the World Cup 2018 in Russia and the World Cup 2014 in Brazil. A total of 128 matches were analyzed using a generalized order logit approach. Twenty-nine variables were identified from previous research. The results showed that defensive errors (*p* = 0.0220), goal efficiency (*p* = 0.0000), duel success (*p* = 0.0000), tackles success (*p* = 0.0100), shots from counterattacks (*p* = 0.0460), clearances (*p* = 0.0130), and crosses (*p* = 0.0160) have a significant influence on winning a match during those tournaments. Ball possession, distance, and market value of the teams had no influence on success. Overall, most of the critical success factors and those with the highest impact on winning close games were defensive actions. Moreover, the results suggest that direct play and pressing were more effective than ball possession play. The study contributes to a better understanding of success factors and can help to improve effectiveness of training, match preparation, and coaching.

## Introduction

To understand the mechanisms underlying success in football is critical for coaches, players, managers, journalists, and other stakeholders. This understanding is in football as crucial as in other sports, but it is still a challenge to determine what leads to success in football. Various attempts have been undertaken to identify and quantify indicators of performance, but results vary and are partly inconsistent. Most studies focused on domestic leagues consisting of club teams, while studies on the performance of national teams at tournaments are sparse. Only 11 studies involving data of success factors from a World Cup were published in recent years (Lepschy et al., 2018). Of these studies, only six used a predictive study design compared with 24 studies about club teams, which can provide more sophisticated conclusions (Lepschy et al., 2018). None of those studies about World Cup matches used market value as an independent variable. Moreover, in most studies, only a small selection of variables was used, and a possible effect of the home advantage was not always considered. Therefore, more research is needed to close this gap in possible unidentified success factors.

Besides a careful consideration of independent variables, a meaningful dependent variable needs to be selected. Regarding the independent variables the most studied variables with regard to success factors in football are shots and shots on goals followed by variables like goal efficiency (number of goals divided by shots), passing, and possession (Sarmento et al., 2014; Lepschy et al., 2018). Goal efficiency and shots on goal were shown to be important factors for winning a football match (Hughes and Franks, 2005; Brito de Souza et al., 2019; Lepschy et al., 2020). Broich et al. (2014) found that goal efficiency is more important than the quantity of shots. They also showed a stronger correlation between goals and goal efficiency than between goals and number of shots. Additionally, Lago-Peñas et al. (2010) found that effectiveness was significantly higher for winning teams. Castellano et al. (2012) showed that the effectiveness of the attacking play was discriminating between winning and losing in three World Cup tournaments (2002–2010). Sarmento et al. (2018) showed in an analysis that fast attacks and counterattacks also increased the success of an attack.

However, ball possession and passing showed mixed results but seem to be not significant success factors if studies are controlled for other variables (Oberstone, 2009; Liu et al., 2015; Lepschy et al., 2020). Notably, Collet (2013) showed that, if controlled for team quality and home advantage, ball possession was a consistent negative effect in domestic leagues as well as club tournaments. On the other hand, Lago-Peñas et al. (2011) came to the conclusion that higher ball possession is a significant influence on winning.

Lepschy et al. (2020) studied the success factors of the German Bundesliga and showed that defensive errors are an influential success factor. They also revealed a significant effect for the total market value of the starting formation. Home advantage and the quality of opponent are two further important contextual variables explaining success (Clarke and Norman, 1995; Santos et al., 2017).

Not only the selection of the independent variables needs to be done carefully, but also the dependent variable needs to be chosen sensibly. Success in football games is usually evaluated based on results (win, draw, and loss) or based on goals (goals scored and conceded). Despite providing more information, the goal-based approach does not perform better than the results-based approach (Goddard, 2005). An alternative method is the approach of the closeness/balance of the game, which allows to overcome the moderator effect that one team does not play at its best level when the game is seemingly decided (Liu et al., 2015; Lepschy et al., 2020). The approach of unbalanced matches and close matches divides the sample into a group of matches with a narrow goal difference (close matches) and a group of matches with a wide goal difference (unbalanced matches). This approach will be also used in this study to reduce data bias and to show success factors that lead to a win in those close matches.

The goal of this exploratory study is to identify the success factors for the FIFA World Cup 2018 in Russia and the FIFA World Cup 2014 in Brazil using an elaborated statistical approach. Twenty-nine variables will be investigated using a results-based approach. This will be the first study to include market value as a success factor of a FIFA World Cup.

## Methods

### Sample

The data used for this study were freely available. Most data (except duel success, distance, average age, and market value) for all 128 matches were collected from www.whoscored.com. The data for duel success were gathered from www.kicker.de. The data on both websites are provided by OPTA. The reliability of the OPTA data lies between 0.92 and 0.94 (Liu et al., 2013). A sample of the collected data has been compared with the official match sheets of the FIFA. The agreement was constantly above 0.9. The data for distance covered were collected from www.fifa.com. The data about market value of the starting formation and average age were retrieved from www.transfermarkt.de, which are provided for each match prior to the kickoff. To take into account the effect of home advantage (12 matches were played by the host nations in 2014 and 2018), a binary dummy variable for home advantage was included in the analysis. To control for the strength of the opponent, the last FIFA coefficient prior to the tournament was used (FIFA, 2014, 2018). Eventually, the 29 variables related to goal scoring, to passing and organizing, to defense, and to context were included in the analysis (Table 1). The tournament rules allow matches to be only decided after 30 min of extra time and/or a penalty shootout. Eight matches were decided through a penalty shootout; these were counted as tied. Five matches were decided after extra time; these were counted as a win for the respective team. The dependent variable was in all cases the results-based outcome of the match, described as win, draw, or loss.

**Table 1:** Performance variables and contextual variables.

**Group**	**Variables**
Variables related to goal scoring	Total shots, shots on target, shots from counterattack, shots from inside 6-yard box, shots from inside penalty area, goal efficiency (Goals × 100/Total shots)
Variables related to passing and organizing	Ball possession (%), passes, pass accuracy (%), long passes, short passes^*a*^, Average pass streak, crosses, successful dribbles, corners, aerials won, distance in kilometers
Variables related to defense	Tackles success (%), fouls, yellow cards, red cards, defensive errors, duel success (%), clearances, interceptions
Contextual variables	Quality of opponent (FIFA coefficient), average age starting formation, total market value starting formation, home advantage (0;1)

a *Removed after test of multicollinearity*.

### Operational Definition

The market value is an estimated figure, which is built on different aspects. The following factors are part of the estimation: performance and stability of the performance, experience, perspectives for the future, and prestige. The market value data have been used in various studies and are considered to be reliable (Göke et al., 2014) and show a high correlation with actual values (Frick, 2011). The average age of the starting formation is the age of each player at the day of the match day summarized and divided by 11. The operational definition of the 25 performance variables can be found on https://www.optasports.com/insight-for-fans/opta-s-event-definitions/ (OPTA, 2018).

### Statistical Analysis

A *K*-means cluster was used to determine the balance of the game. One hundred eight matches were classified as close (goal difference 0–2 goals) and 20 matches as unbalanced (goal difference 3 or more goals). The 108 matches were analyzed twice, since the home team (first mentioned team) on the schedule is not playing at home except for the 12 matches mentioned before. Hence, this analysis is based on 216 observations.

The test of parallel regression was significant (Brant: chi^2^ = 260.7; *p* = 0.000); therefore, the assumption of proportional odds is violated (Brant, 1990). Consequently, the generalized ordered logit regression was used for the analysis (Williams, 2016). To test for the multicollinearity, the command *collin* was used (Ender, 2010). A variance inflation factor (VIF) above 10 was set as the cutoff value (Craney and Surles, 2002). The variables passes (VIF = 654.69) and short passes (VIF = 662.10) showed higher values. The variable short passes was removed from the model. Pseudo *R*^2^ of the analyzed model was 0.3622.

To interpret the results, marginal effects (command *margins*) were calculated (Williams, 2012). The significance level was set to *p* < 0.05 for all statistical analyses.

The data were analyzed in IBM SPSS Statistics 24 and STATA 15. The study received ethical approval by the Institutional Review Board of the Institute of Sports and Sports Science, Karlsruhe, Germany.

## Results

The descriptive statistics are presented in Table 2. The average goals per match were 2.66 (2.64 in 2018 and 2.67 in 2014).

**Table 2:** Descriptive statistics.

	**Mean**	**Std. deviation**	**Std. error**	**95% confidence interval for mean**		**Minimum**	**Maximum**
				**Lower bound**	**Upper bound**		
Total shots	13.00	5.43	0.37	12.27	13.72	3.00	39.00
Shots on target	4.14	2.41	0.16	3.82	4.47	0.00	17.00
Shots from counterattack	0.37	0.80	0.05	0.26	0.48	0.00	5.00
Shots from inside 6-yard box	0.73	0.92	0.06	0.60	0.85	0.00	4.00
Shots from inside penalty area	6.40	3.34	0.23	5.96	6.85	1.00	23.00
Goal efficiency	10.12	9.68	0.66	8.83	11.42	0.00	57.14
Ball possession (%)	50.00	12.46	0.85	48.33	51.67	21.00	79.00
Passes	447.32	137.83	9.38	428.83	465.80	156.00	*1, 115*.00
Pass accuracy (%)	79.97	7.10	0.48	79.02	80.92	57.00	93.00
Long passes	58.92	13.96	0.95	57.05	60.79	29.00	107.00
Short passes	434.05	143.17	9.74	414.85	453.25	147.00	*1, 104*.00
Average pass streak	4.61	1.28	0.09	4.44	4.78	2.00	10.00
Crosses	18.78	8.69	0.59	17.61	19.94	3.00	53.00
Successful dribbles	10.14	4.62	0.32	9.52	10.76	1.00	23.00
Corners	5.07	2.75	0.19	4.71	5.44	0.00	19.00
Aerials won	17.57	7.59	0.52	16.55	18.58	2.00	49.00
Distance	109.63	11.73	0.80	108.05	111.20	93.00	155.00
Tackles success (%)	64.63	10.76	0.73	63.19	66.08	33.33	94.44
Fouls	14.26	5.03	0.34	13.58	14.93	4.00	31.00
Yellow cards	1.57	1.15	0.08	1.42	1.73	0.00	6.00
Red cards	0.05	0.21	0.01	0.02	0.08	0.00	1.00
Defensive errors	0.40	0.65	0.04	0.31	0.49	0.00	3.00
Duel success (%)	50.00	5.46	0.37	49.27	50.73	36.00	64.00
Clearances	25.25	10.68	0.73	23.82	26.68	4.00	67.00
Interceptions	11.66	5.00	0.34	10.99	12.33	2.00	29.00
FIFA coefficient	964.53	249.78	17.00	931.03	998.03	457.00	*1, 558*.00
Average age starting formation	27.84	1.38	0.09	27.65	28.02	24.40	30.90
Total market value starting formation	191.52	180.41	12.28	167.32	215.71	4.83	710.00

The marginal effects for the outcome “win” of all analyzed variables are displayed in Table 3. Shots from counterattack, goal efficiency, crosses, tackle success (%), defensive errors, duel success (%), and clearances had a significant influence on winning a match. Defensive errors showed the highest influence (dy/dx = −0.1025, *p* < 0.05), with one defensive error decreasing the probability of winning by 10.25%. One additional shot from a counterattack increased the chance of winning by 6.51% (dy/dx = 0.0651, *p* < 0.05). However, duel success (%) and goal efficiency showed to be important as well and highly significant (dy/dx = 0.0214, *p* < 0.01, respectively, dy/dx = 0.0193, *p* < 0.01). None of the contextual variables showed a significant impact. However, the contextual variable home advantage had the highest positive value (dy/dx = 0.0822, *p* = 0.4780) of all variables^1^.

**Table 3:** Marginal effects for the outcome “win.”

	**dy/dx**	**Std. err**.	***z***	***p* > *z***	**95% conf. interval**	
Total shots	0.0149	0.0099	1.5100	0.1310	−0.0044	0.0343
Shots on target	0.0182	0.0153	1.1900	0.2340	−0.0118	0.0481
Shots from counterattack*	0.0651	0.0326	2.0000	0.0460	0.0012	0.1291
Shots from inside 6-yard box	0.0090	0.0278	0.3200	0.7460	−0.0454	0.0634
Shots from inside penalty area	0.0003	0.0124	0.0200	0.9810	−0.0239	0.0245
Goal efficiency**	0.0193	0.0034	5.7300	0.0000	0.0127	0.0259
Ball possession (%)	0.0091	0.0052	1.7500	0.0810	−0.0011	0.0192
Passes	−0.0004	0.0005	−0.7600	0.4450	−0.0014	0.0006
Pass accuracy (%)	−0.0082	0.0065	−1.2700	0.2050	−0.0209	0.0045
Long passes	−0.0013	0.0022	−0.5700	0.5710	−0.0057	0.0031
Average pass streak	0.0121	0.0393	0.3100	0.7590	−0.0650	0.0891
Crosses*	−0.0111	0.0046	−2.4100	0.0160	−0.0201	−0.0021
Successful dribbles	−0.0066	0.0065	−1.0300	0.3050	−0.0193	0.0060
Corners	0.0044	0.0127	0.3400	0.7320	−0.0205	0.0293
Aerials won	−0.0021	0.0036	−0.5900	0.5540	−0.0092	0.0049
Distance	−0.0021	0.0028	−0.7600	0.4470	−0.0075	0.0033
Tackles success (%)*	0.0057	0.0022	2.5600	0.0100	0.0013	0.0100
Fouls	0.0065	0.0065	1.0000	0.3150	−0.0062	0.0192
Yellow cards	−0.0148	0.0192	−0.7700	0.4400	−0.0525	0.0228
Red cards	−0.0165	0.0768	−0.2100	0.8300	−0.1669	0.1339
Defensive errors*	−0.1025	0.0448	−2.2900	0.0220	−0.1903	−0.0148
Duel success (%)**	0.0214	0.0062	3.4900	0.0000	0.0094	0.0335
Clearances*	0.0084	0.0034	2.4900	0.0130	0.0018	0.0150
Interceptions	−0.0038	0.0046	−0.8300	0.4080	−0.0130	0.0053
FIFA coefficient	0.0002	0.0001	1.3600	0.1730	−0.0001	0.0004
Average age starting formation	−0.0190	0.0171	−1.1100	0.2660	−0.0525	0.0145
Total market value starting formation	0.0001	0.0002	0.5300	0.5980	−0.0003	0.0005
Home advantage	0.0822	0.1158	0.7100	0.4780	−0.1448	0.3093

**p < 0.001; *p < 0.05.

**Figure 1:**
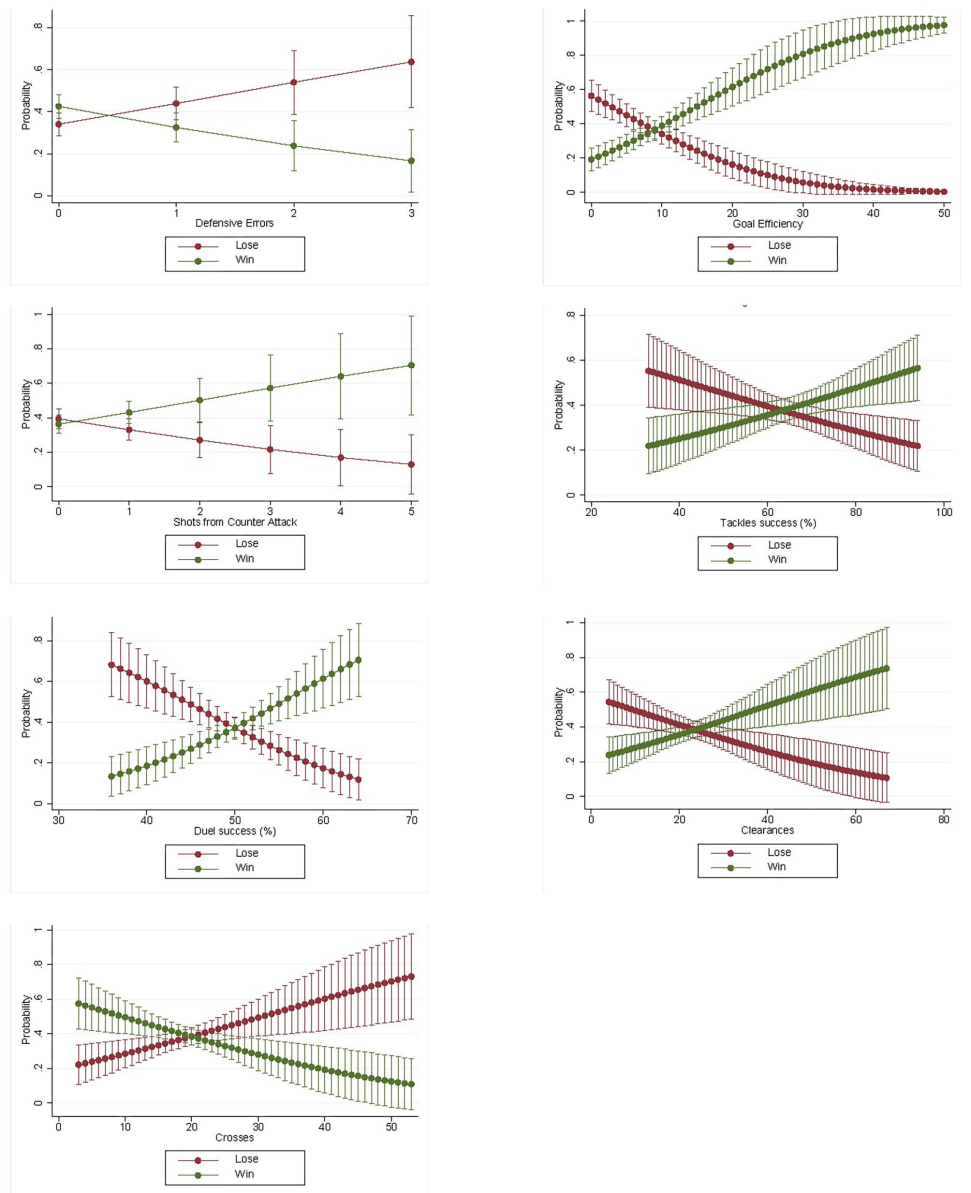
Margins with 95% CIs of the significant variables.

The seven significant variables including the 95% confidence intervals are also shown in Figure 1. All graphs show a clear development of the predictors regarding the probability of winning or losing. The higher or lower the value of the predictor, the higher is the probability of winning or losing.

## Discussion

The purpose of this research was to identify success factors in the games played at the Football World Cups in 2018 and 2014. The significant positive success factors during the World Cup 2018 and 2014 were shots from counterattack, duel success (%), goal efficiency (%), clearances and tackles success (%). On the other hand, defensive errors and crosses had a significant negative impact on the probability of winning. Despite that none of the contextual factors in this study were significant, it is still worth noting that the effects of those variables were substantial.

### Overview of Significant Variables

Of the significant variables, four variables related to defense [defensive errors, tackles success (%), duel success (%), and clearances] were significant. Two variables related to goal scoring [goal efficiency (%) and shots from counterattack], and one variable related to passing and organizing (crosses) showed significant influence. No contextual variables were significant.

### Significant Defensive Factors

The most influential success factor was defensive errors. Each defensive error decreases the probability of winning by 10.25% (*p* < 0.001). Despite being an intuitive result, defensive errors were rarely analyzed in recent studies, and this study permits a quantification of the impact. Lepschy et al. (2020) showed similar results for the German Bundesliga. The impact of errors in this study is slightly higher than the impact in the German Bundesliga. The operational definition of a defensive errors could also contribute to the big impact, “A mistake made by a player losing the ball that leads to a shot or a goal” (OPTA, 2018). Losing the ball by a mistake usually also leaves the defense in an imbalanced status. Tenga et al. (2010) showed that playing against an imbalanced defense increases the chance of a goal for the attacking team. Several studies showed that the chance of a defensive errors is also increasing toward the end of a match because of physical deterioration and diminished cognitive function (Simiyu, 2014). The next significant factor related to defense is duel success in percentage, showing the third highest value of all significant success factors. Each additional percentage increases the chance of winning by 2.14% (*p* < 0.001). However, duel success has the lowest standard deviation of the following significant defensive factors and has the lowest range. Therefore, it could be argued that despite the higher value, the positive effect of duel success is limited. Furthermore, the percentage of successful tackles was a significant positive success factor as well (0.57%, *p* < 0.05). However, previous research has yielded inconclusive results about whether the percentage of successful tackles is significant or not (Oberstone, 2009; Liu et al., 2015, 2016). Future research should investigate this further and focus on identifying possible interacting factors such as the location of the tackles or the direction of the tackles. Finally, clearances showed a significant positive effect (0.84%, *p* < 0.05) on success. This confirms previous research by Carmichael et al. (2000) and Lepschy et al. (2020). However, clearances were only rarely included in past research. In the light of those results, future research should consider including clearances for an analysis of success factors in football.

### Significant Offensive Factors

Besides the multitude of significant defensive factors, the analysis revealed that there are also actions of offensive performance that can make the difference. Notably, each shot from a counterattack increased the chance of winning by 6.51% (*p* < 0.05). Moreover, the conversion of shots into goals is shown to be a very important success factor. In agreement with previous research, it was shown that goal efficiency has a significant positive effect on winning (Broich et al., 2014; Lepschy et al., 2020). A positive change of one percentage in goal efficiency increases the chance of winning by 1.93% (*p* < 0.001).

### Significant Factors Related to Passing and Organizing

Crosses are the only significant variable related to passing and organizing. The number of crosses had a significant negative effect (−1.11%, *p* < 0.05). Again, this confirms previous research (Lago-Peñas et al., 2010; Liu et al., 2015; Lepschy et al., 2020). The reason might be that only quantity and not quality of crosses was considered. This assumption is supported by a study that found that long passes are linked to losing ball possession (Reis et al., 2017). Unsuccessful crosses are likely to initiate a counterattack. Moreover, crosses from the midfield could be an indicator of limited technical and tactical skills or a compact defense of the opponent. Nevertheless, there is also an indication of a positive effect for crosses (Oberstone, 2009). Hence, future research should consider the quality of crosses.

### Non-significant Factors in Contrast to Previous Research

The effect of ball possession has been discussed controversially. It was not a significant predictor in past FIFA tournaments if other variables were included in the model (Collet, 2013). However, studies related to success factors in football leagues show ambiguous results (Collet, 2013; Liu et al., 2015, 2016). In this study, ball possession showed no effect, supporting the assumption that ball possession is losing significant impact if the results are controlled for other influencing variables (Collet, 2013).

Interestingly, total shots, shots from inside six-yard box, and shots from inside penalty area did not affect the outcome of the games. Total shots and subgroups of shots (shots from inside six-yard box and shots from inside penalty area) were widely studied in the past, and the results showed mostly a significant positive effect on success (Oberstone, 2009; Lago-Peñas et al., 2010; Liu et al., 2015, 2016; Pappalardo and Cintia, 2018; Lepschy et al., 2020). However, our non-significant results might be due to goal efficiency and points toward the importance of precision over quantity of shots.

In our analysis, distance showed no effect, although recent studies identified it as the most influential variable in the German Bundesliga (Schauberger et al., 2017). However, in the latest study on the Bundesliga, with a wide range of variables, distance had also no effect on success (Lepschy et al., 2020).

In contradiction to prior results of the German Bundesliga, market value was not a significant predictor of success (Lepschy et al., 2020). Seemingly, the market value of national teams at World Cups is less important than in club football. A reason might be the different character of tournament games including single knockout games to games played during a regular season. However, further research is needed to determine if this hypothesis can be supported. Other explanations could be that not enough matches with a distinct difference in market value were included or that a mediator variable, which is not yet identified, is present.

In general, it showed that actions related to defense had a high impact of success in the last two World Cups. Moreover, it appears that variables related to efficiency such as duel success (%), goal efficiency (%), and tackles success (%) are more important than the quantity of single factors, a finding that is supported by Collet (2013). Finally, ball possession seems to be of less importance also on a national team level. A more pressing/direct style, as reflected in defensive errors of the opponent and shots from counterattacks as well as duel and tackles success, seems to be more successful. This finding is in line with other studies (Pollard, 2019).

### Practical Implications

The results of this study have various implications for coaches of national teams but could also be helpful for coaches of club teams. Our findings point toward aspects that can make the difference at high-level football matches. Shot accuracy during matches is critical and should be properly addressed in training sessions. The development and utilization of apt training methods could be beneficial for the goal efficiency and eventually lead to more success. Accuracy instead of quantity should be the maxim. Furthermore, more effective ways to lower the probability of defensive errors should be found and implemented in specific training sessions. Next to technical and tactical skills, the improvement of endurance, speed, and mental strength could be critical in this context. To increase the duel and tackles success rate, specific training methods could be utilized, and players should be focused on the importance of these factors in match preparations. Substitutions to accommodate for physical and mental fatigue of the starting formation could also contribute to a lower error rate and can help to win a match [see also Njororai (2012) and Njororai (2013)]. Instead of substituting forwards in during the second half, coaches could consider strengthening the defense through specific substitutions. On the tactical side, coaches should be aware of the significance of counterattacks especially when playing against stronger opponents. The play against an imbalanced defense can lead to more scoring opportunities especially if played at a faster pace (Almeida, 2019).

### Limitations and Future Research

By interpreting the results of this study, four restrictions have to be taken into account. First, the sample size contained only matches of national teams during a tournament including only 128 matches. Therefore, the possible generalization of the results is limited. In addition, the sample consisted of matches from the group stages and knockout stages. The tactics used in the different stages could have interfered with the results. Second, the variable short passes was dropped in favor of reduced collinearity. Any effects of this variable were not accounted for. Third, the variable market value of the starting formation was gathered from a public website and is not a standardized factor. Fourth, the data were collected from third parties, and the reliability of their collection process was checked in previous research for a specific sample, which showed 0.92 and 0.94 and not directly for the obtained samples (see Sample section).

With regard to future research, the study points toward several aspects that need further investigation. The influence of ball possession needs to be analyzed in more detail. This study showed no significant influence, which is in agreement with previous research (Collet, 2013; Lepschy et al., 2020). However, other recent studies found a significant effect of ball possession but in opposite directions (Liu et al., 2015; Schauberger et al., 2017).

Future research also needs to analyze the effects of the distance covered, since results are inconsistent. In addition, the negative impact of crosses should be analyzed. Lepschy et al. (2020) found similar results for the Bundesliga. It needs to be determined when crosses are a negative predictor and in which cases they are not. Moreover, the non-significant influence of shots, except shots from counterattack, should be investigated further to confirm previous results that showed a clear positive effect (Oberstone, 2009; Lago-Peñas et al., 2010; Liu et al., 2015; Pappalardo and Cintia, 2018; Brito de Souza et al., 2019; Lepschy et al., 2020). Also, the effect of home advantage at World Cups needs to be studied further considering crowd support, climate, and possible influences of a “once in a lifetime experience” for players. Additionally, the styles of play could be incorporated into a model of success factors. Not only does the match status interact with the playing style, but also certain performance factors can be linked to a specific style. The success factors could vary between those playing styles and contribute to a better understanding of the mechanism of success in football (Courneya and Cheiadurai, 1991; Fernandez-Navarro et al., 2016, 2018; Hewitt et al., 2016; Lago-Peñas et al., 2017; Gómez et al., 2018; Castellano and Pic, 2019).

Methodologically, predictive analyses are the methods of choice. However, alternative methodological approaches such as social network analysis (Wäsche et al., 2017) should be considered. Social network analysis already revealed some new insights (Pina et al., 2017).

## Conclusion

The study showed that defensive errors had the strongest influence on the probability of winning or losing a football match during the World Cups 2018 and 2014. In addition, goal efficiency, duel success in percentage, and tackles success in percentage were shown to be of high significance. It appears that efficiency factors are more important than single factors alone. Shots from counterattacks and clearances also revealed a positive impact. In contrast, the number of crosses showed a negative impact on winning. In total, four different variables related to defense, two variables related to goal scoring, one variable related to passing and organizing, and no contextual variables were significant. Interestingly, shots from counterattacks, tackles, and duel success are significant predictors of success, whereas ball possession and passes are not significant. This could be an indicator for the assumption that tactics dominated by pressing could be a better strategy than tactics solely based on ball possession. However, national teams and club teams cannot readily be compared due to different contexts such as the competition format. Future research needs to determine possible differences. In addition, the ambiguous results for ball possession and number of crosses from different studies needs to be addressed in future research. Further research on success factors, building on existing knowledge and utilizing apt methods, will further contribute to the knowledge of coaches, managers, and other practitioners to improve team performance in football.

## Data Availability Statement

The datasets generated for this study are available on request to the corresponding authors.

## Ethics Statement

Studies involving animal subjects: No animal studies are presented in this manuscript. Studies involving human subjects: No human studies are presented in this manuscript. Inclusion of identifiable human data: No potentially identifiable human images or data is presented in this study.

## Author Contributions

HL contributed to the conception and design of the study, the data collection, the data analysis and interpretation, the drafting the article, and gave the final approval of the version to be published. HW and AW contributed to the conception and design of the study, the data analysis and interpretation, the drafting the article, and gave the final approval of the version to be published. All authors contributed to the article and approved the submitted version.

## Conflict of Interest

The authors declare that the research was conducted in the absence of any commercial or financial relationships that could be construed as a potential conflict of interest.

## Footnotes

^1^Non-significance is due to the small sample size for home advantage (*n* = 12).

## References

[B1] Almeida C. H. (2019). Comparison of successful offensive sequences in the group stage of 2018 FIFA World Cup. Eliminated vs. qualified teams. Sci. Med. Football 1, 1–7. 10.1080/24733938.2019.1613557

[B2] Brant R. (1990). Assessing proportionality in the proportional odds model for ordinal logistic regression. Biometrics 46, 1171–1178. 10.2307/2532457 2085632

[B3] Brito de Souza D. López-Del Campo R. Blanco-Pita H. Resta R. Del Coso J. (2019). An extensive comparative analysis of successful and unsuccessful football teams in LaLiga. Front. Psychol. 10:2566. 10.3389/fpsyg.2019.02566 31781011 PMC6856952

[B4] Broich H. Mester J. Seifriz F. Zengyuan Y. U. E. (2014). Statistical analysis for the first Bundesliga in the current soccer season. Adv. Appl. Math. 7, 1–8.

[B5] Carmichael F. Thomas D. Ward R. (2000). Team performance. The case of English Premiership football. Manag. Decis. Econ. 21, 31–45. 10.1002/1099-1468(200001/02)21:1<31::AID-MDE963>3.0.CO;2-Q

[B6] Castellano J. Casamichana D. Lago C. (2012). The use of match statistics that discriminate between successful and unsuccessful soccer teams. J. Hum. Kinet. 31, 139–147. 10.2478/v10078-012-0015-7 23487020 PMC3588662

[B7] Castellano J. Pic M. (2019). Identification and preference of game styles in LaLiga associated with match outcomes. Int. J. Environ. Res. Public Health 16, 5090. 10.3390/ijerph16245090 31847147 PMC6950299

[B8] Clarke S. R. Norman J. M. (1995). Home ground advantage of individual clubs in English soccer. Statistician 44, 509–521. 10.2307/2348899

[B9] Collet C. (2013). The possession game? A comparative analysis of ball retention and team success in European and international football, 2007-2010. J. Sports Sci. 31, 123–136. 10.1080/02640414.2012.727455 23067001

[B10] Courneya K. S. Cheiadurai P. (1991). A model of performance measures in baseball. J. Sport Exerc. Psychol. 13, 16–25. 10.1123/jsep.13.1.16

[B11] Craney T. A. Surles J. G. (2002). Model-dependent variance inflation factor cutoff values. Qual. Eng. 14, 391–403. 10.1081/QEN-120001878

[B12] Ender P. B. (2010). Collin. Collinearity Diagnostics. Institute for Digital Research and Education, University of California, Los Angeles.

[B13] Fernandez-Navarro J. Fradua L. Zubillaga A. Ford P. R. McRobert A. P. (2016). Attacking and defensive styles of play in soccer: analysis of Spanish and English elite teams. J. Sports Sci. 34, 2195–2204. 10.1080/02640414.2016.1169309 27052355

[B14] Fernandez-Navarro J. Fradua L. Zubillaga A. McRobert A. P. (2018). Influence of contextual variables on styles of play in soccer. Int. J. Perform. Anal. Sport 18, 423–436. 10.1080/24748668.2018.1479925

[B15] FIFA (2014). FIFA World Ranking. Available online at: https://www.fifa.com/fifa-world-ranking/ranking-table/men/rank=239/index.html (accessed August 05, 2018).

[B16] FIFA (2018). FIFA World Ranking. Available online at: https://www.fifa.com/fifa-world-ranking/ranking-table/men/rank=287/index.html (accessed August 05, 2018).

[B17] Frick B. (2011). Performance, salaries and contract length: empirical evidence from German Soccer. Int. J. Sport Finan. 6, 87–118. Available online at: https://fitpublishing.com/content/performance-salaries-and-contract-length-empirical-evidence-german-soccer-pp-87-118

[B18] Goddard J. (2005). Regression models for forecasting goals and match results in association football. Int. J. Forecast. 21, 331–340. 10.1016/j.ijforecast.2004.08.002

[B19] Göke S. Prinz J. Weimar D. (2014). Diamonds are forever: job-matching and career success of young workers. J. Econ. Stat. 234, 450–473. 10.1515/jbnst-2014-0402

[B20] Gómez M.-Á. Mitrotasios M. Armatas V. Lago-Peñas C. (2018). Analysis of playing styles according to team quality and match location in Greek professional soccer. Int. J. Perform. Anal. Sport 18, 986–997. 10.1080/24748668.2018.1539382

[B21] Hewitt A. Greenham G. Norton K. (2016). Game style in soccer: what is it and can we quantify it? Int. J. Perform. Anal. Sport. 16, 355–372. 10.1080/24748668.2016.11868892

[B22] Hughes M. Franks I. (2005). Analysis of passing sequences, shots and goals in soccer. J. sport sci. 23, 509–514. 10.1080/02640410410001716779 16194998

[B23] Lago-Peñas C. Gómez-Ruano M. Yang G. (2017). Styles of play in professional soccer: an approach of the Chinese Soccer Super League. Int. J. Perform. Anal. Sport. 17, 1073–1084. 10.1080/24748668.2018.1431857

[B24] Lago-Peñas C. Lago-Ballesteros J. Dellal A. Gómez M. (2010). Game-related statistics that discriminated winning, drawing and losing teams from the Spanish soccer league. J. Sports Sci. Med. 9, 288–293. Available online at: https://www.jssm.org/jssm-09-288.xml%3EFulltext 24149698 PMC3761743

[B25] Lago-Peñas C. Lago-Ballesteros J. Rey E. (2011). Differences in performance indicators between winning and losing teams in the UEFA Champions League. J. Hum. Kinet. 11, 32–41. 10.2478/v10078-011-0011-3

[B26] Lepschy H. Wäsche H. Woll A. (2018). How to be successful in football. A Systematic Review. Open Sports Sci. J. 11, 3–23. 10.2174/1875399X01811010003

[B27] Lepschy H. Wäsche H. Woll A. (2020). Success factors in football: an analysis of the German Bundesliga. Int. J. Perform. Anal. Sport 20, 150–164. 10.1080/24748668.2020.1726157

[B28] Liu H. Gomez M.-Á. Lago-Peñas C. Sampaio J. (2015). Match statistics related to winning in the group stage of 2014 Brazil FIFA World Cup. J. Sports Sci. 33, 1205–1213. 10.1080/02640414.2015.1022578 25793661

[B29] Liu H. Hopkins W. Gómez A. M. Molinuevo S. J. (2013). Inter-operator reliability of live football match statistics from OPTA Sportsdata. Int. J. Perform. Anal. Sport 13, 803–821. 10.1080/24748668.2013.11868690

[B30] Liu H. Hopkins W. G. Gómez M.-A. (2016). Modelling relationships between match events and match outcome in elite football. Eur. J. Sport Sci. 16, 516–525. 10.1080/17461391.2015.1042527 26190577

[B31] Njororai W. (2012). Physical demands of soccer: Lessons from team USA and Ghana matches in the 2010 FIFA WORLD CUP. J. Phys. Educ. Sport 12, 407–412. 10.7752/jpes.2012.04060

[B32] Njororai W. W. S. (2013). Analysis of goals scored in the 2010 world cup soccer tournament held in South Africa. J. Phys. Educ. Sport 13, 6–13. 10.7752/jpes.2013.01002

[B33] Oberstone J. (2009). Differentiating the Top English Premier League Football Clubs from the Rest of the Pack. Identifying the Keys to Success. J. Quant. Anal. Sports 5, 10. 10.2202/1559-0410.1183

[B34] OPTA (2018). Opta's Event Definitions. Available online at: https://www.optasports.com/insight-for-fans/opta-s-event-definitions/ (accessed January 16, 2021).

[B35] Pappalardo L. Cintia P. (2018). Quantifiying the relation between Performance and success in soccer. Adv. Complex Syst. 21, 1750014. 10.1142/S021952591750014X

[B36] Pina T. J. Paulo A. Araújo D. (2017). Network characteristics of successful performance in association football. A Study on the UEFA Champions League. Front. Psychol. 8:534. 10.3389/fpsyg.2017.0117328450838

[B37] Pollard R. (2019). Invalid interpretation of passing sequence data to assess team performance in football: repairing the tarnished legacy of charles reep. Open Sports Sci. J. 12, 17–21. 10.2174/1875399X01912010017

[B38] Reis M. A. M. d Vasconcellos F. V. d A Almeida M. B. d. (2017). Analysis of the effectiveness of long distance passes in 2014 Brazil FIFA World Cup. Rev. Bras. Cineantropom. Desempenho Hum. 19, 676–685. 10.5007/1980-0037.2017v19n6p676

[B39] Santos P. Lago-Peñas C. García-García O. (2017). The influence of situational variables on defensive positioning in professional soccer. Int. J. Perform. Anal. Sport 17, 212–219. 10.1080/24748668.2017.1331571

[B40] Sarmento H. Figueiredo A. Lago-Peñas C. Milanovic Z. Barbosa A. Tadeu P.. (2018). Influence of tactical and situational variables on offensive sequences during elite football matches. J. Strength Cond. Res. 32, 2331–2339. 10.1519/JSC.0000000000002147 28737587

[B41] Sarmento H. Marcelino R. Anguera M. T. CampaniÇo J. Matos N. LeitÃo J. C. (2014). Match analysis in football. A systematic review. J. Sports Sci. 32, 1831–1843. 10.1080/02640414.2014.898852 24787442

[B42] Schauberger G. Groll A. Tutz G. (2017). Analysis of the importance of on-field covariates in the German Bundesliga. J. Appl. Stat. 11, 1–18. 10.1080/02664763.2017.1383370

[B43] Simiyu W. W. N. (2014). Timing of goals scored in selected european and South American Football Leagues, FIFA and UEFA tournaments and the critical phases of a match. Int. J. Sports Sci. Coach 4, 56–64. 10.5923/s.sports.201401.08

[B44] Tenga A. Holme I. Ronglan L. T. Bahr R. (2010). Effect of playing tactics on goal scoring in Norwegian professional soccer. J. Sports Sci. 28, 237–244. 10.1080/02640410903502774 20391095

[B45] Wäsche H. Dickson G. Woll A. Brandes U. (2017). Social network analysis in sport research. An emerging paradigm. Eur. J. Sport Soc. 14, 138–165. 10.1080/16138171.2017.1318198

[B46] Williams R. (2012). Using the margins command to estimate and interpret adjusted predictions and marginal effects. Stata J. 12, 308. 10.1177/1536867X1201200209

[B47] Williams R. (2016). Understanding and interpreting generalized ordered logit models. J. Math. Sociol. 40, 7–20. 10.1080/0022250X.2015.1112384

